# Public Rental Housing and Obesogenic Behaviors among Adults in Hong Kong: Mediator Role of Food and Physical Activity Environment

**DOI:** 10.3390/ijerph19052960

**Published:** 2022-03-03

**Authors:** Ting Zhang, Bo Huang, Hung Wong, Samuel Yeung-shan Wong, Roger Yat-Nork Chung

**Affiliations:** 1 School of International and Public Affairs, Shanghai Jiao Tong University, Shanghai 200030, China; tingzhang@sjtu.edu.cn; 2 Institute of Space and Earth Information Science, The Chinese University of Hong Kong, Hong Kong 999077, China; 3 Department of Geography and Resource Management, The Chinese University of Hong Kong, Hong Kong 999077, China; 4 Shenzhen Research Institute, The Chinese University of Hong Kong, Shenzhen 518057, China; 5 Department of Social Work, The Chinese University of Hong Kong, Hong Kong 999077, China; hwong@cuhk.edu.hk; 6 CUHK Institute of Health Equity, The Chinese University of Hong Kong, Hong Kong 999077, China; yeungshanwong@cuhk.edu.hk (S.Y.-s.W.); rychung@cuhk.edu.hk (R.Y.-N.C.); 7 Jockey Club School of Public Health and Primary Care, The Chinese University of Hong Kong, Hong Kong 999077, China

**Keywords:** physical inactivity, prolonged sitting, unhealthy diet, sports facilities, street greenery

## Abstract

Public rental housing (PRH) for low-income families has been shown in several studies to be associated with poor health status and obesity. However, the causes of this health disparity are controversial, and the associations and pathways between PRH and obesogenic behaviors remain unknown. Using cross-sectional survey data of 1977 adults living in Hong Kong (aged or over 18) together with multi-source GIS-based environmental data, we examined the associations between PRH and obesogenic behaviors and the extent to which those associations can be explained by neighborhood food and physical environment. The unhealthy food environment, which relates with infrequent fruit and vegetables consumption, was calculated based on the relative density of fast food restaurants and convenience stores to grocery stores. The physical activity environment, which relates to physical inactivity and prolonged sitting, was assessed in terms of density of sports facilities and street greenery, separately. Regressions and mediation analyses show that PRH was negatively associated with physical inactivity directly and also indirectly via higher sports facilities density; however, PRH was positively associated with unhealthy diet largely directly and positively associated with prolonged sitting indirectly via less street greenery. We advanced the international literature of PRH health impact assessment and its environmental health pathways by providing evidence from the least housing-affordable city in the world. The findings provide planning implications in formulating a healthier PRH community for these low-income PRH households and mitigating health disparities induced by housing type.

## 1. Introduction

Public rental housing (PRH), one of the most effective housing solutions for low-income families, has been employed in many countries around the world to improve living conditions of those needy families. Goal 11 of the United Nation’s 2030 Agenda for Sustainable Development reflects the significance of inclusive and healthy cities: “making cities inclusive, safe, resilient and sustainable” [1]. Rapid urbanization exerts enormous pressure on housing, due to shortages of land resources and an expanding population. People with low socio-economic status (SES) tend to live in terrible environments. They are more vulnerable to negative health impacts from their living environments [2,3]. The increasing number of slum dwellers in cities and metropolitan areas, especially in Eastern and Southeastern Asia, has led local governments to take actions such as PRH to safeguard the basic human right of adequate housing. The research on the health impacts of PRH is of particular importance in the context of rapid urbanization, especially in Asia.

There is a systematic review of a total of 14 articles, including 4 prospective studies, 8 cross-sectional studies, and 2 retrospective cohort studies on the relationship between staying in PRH in Singapore and health status [4]. Staying in PRH was found to be associated with all-cause mortality in Singapore and increases hospital utilization [4,5]. Similar to the findings in the United States, whereby the low-income housing policy and poor health has been linked for more than a century [6]. Although residing in PRH appears to be a risk marker of poorer health, the causes of health disparity are controversial. The argument is over if residents entered PRH already ill or if PRH may cause the poor health of its resident [7]. A mixed-methods study in Atlanta, USA, found that the majority of PRH residents entered PRH already ill, and the long tenure in PRH was not associated with an increased poor health after entry into PRH [7]. However, another study using a nationally representative survey in the large cities in the USA found that PRH residency increases obesity and worsens mothers’ overall health status by minimizing the selection effect with controls and instrumental variables [6]. The mixed results suggest the urgent need for more research to find out the pathways from residing in PRH to related health outcomes.

From the perspective of the paradigm of traditional housing health research, both interior housing condition and external neighborhood environment could affect residents’ health [8]. If PRH residence leads to poor health outcomes, the pathways could be related with poor housing and neighborhood environment in PRH. However, a related study, which leveraged a natural experiment created by PRH redevelopment in Los Angeles, found that there was no significant change in weight-related outcomes during the two years of baseline after the improvements to housing and the built and social environments in PRH communities [9]. Yet, weight-related outcomes, such as body mass index, are relatively long-term health consequences and need to be observed over time. In addition, they are complex, with multiple possible pathways, including both eating and physical activity. The short-term influences include health-related behaviors, attitudes, and health-seeking behaviors [10].

Therefore, more studies are needed to assess the short-term health impact of PRH residence, such as obesogenic behaviors, to help understand the pathways of the health disparities induced by housing type. Surprisingly, such research is scarce. A natural experimental study aimed to assess whether enhancing the physical and social environment could reduce obesity among PRH residents via improving diet and physical activity or not [11]. From baseline to one-year follow-up, they found that the intervention participants significantly changed their eating and activity behaviors, while control participants reported no significant change in any studied variables [12]. However, the associations and pathways linking PRH residence to obesogenic behaviors still need to be further explored.

In Hong Kong, PRH is the primary housing solution to achieve the vision of helping all households gain access to adequate and affordable housing based on the Long Term Housing Strategy (2014), and it was introduced in 1954, after the Shek Kip Mei fire [13]. Approximately 2.13 million persons (29.1%) live in PRH according to the Hong Kong By-census (2016), and the average waiting time for PRH was 4.7 years in 2016 [14]. PRH is not for sale and provides a safety net for low-income families with the eligibility criteria of income and assets. Some studies explored associations between residential environment and health among Hong Kong’s PRH residents [15,16]. However, whether there were disparities in the residential environment and health behaviors among PRH and non-PRH residence remains unknown in Hong Kong, as Hong Kong has a different PRH governance from Western cites or other Asian areas such as Singapore. Studies on the associations and environmental pathways between PRH residence and health-related outcomes are very meaningful in one of the least housing-affordable cities in the world. Meanwhile, obesity has become a global problem, spreading rapidly across Asia in the setting of urbanization. In Hong Kong, about 30% of adults are obese and 20% are overweight [17].

To fill the above gaps, this paper examined the associations and pathways linking PRH residence to obesogenic behaviors via food and physical activity environment among adults in a Hong Kong context. We used GIS-based environmental variables together with a representative cross-sectional survey data among Hong Kong adults from 2014 to 2015. Two aspects were analyzed with regression and mediation models: (1) associations of PRH with food and physical activity environment and (2) the direct and indirect associations from PRH to obesogenic behaviors via corresponding food and physical activity environmental exposures. This study aims to not only verify the association among PRH, food, and physical activity environments and obesogenic behaviors but also to highlight the importance of neighborhood environments in mitigating health disparities induced by housing type. The findings in this study advanced the international literature of PRH health impact assessment and its environmental health pathways in the context of a representative world city, which provided governance implications for healthy PRH communities.

## 2. Theoretical Foundation and Hypothesis Development

**Figure 1:**
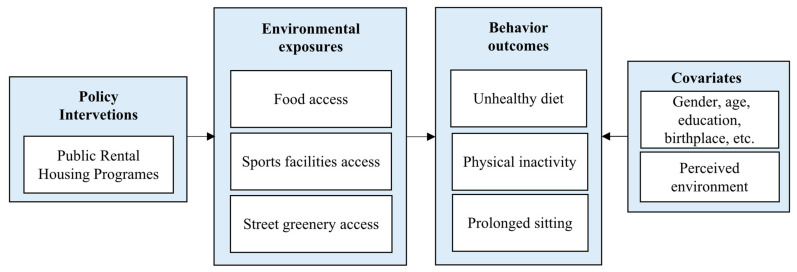
Conceptual framework of direct and indirect pathways through which public rental housing programs influence obesogenic behaviors.

Generally, we assumed that PRH residence is associated with obesogenic behaviors via residential environment (summarized in Figure 1) with three detailed hypotheses in a Hong Kong context: (1) PRH residence → unhealthy food environment → infrequent fruits and vegetables (FV) eating; (2) PRH residence → less sports facility density → less physical inactivity; (3) PRH residence → less street greenery → prolonged sitting. These hypotheses include two parts: (1) PRH residence → neighborhood environmental exposures and (2) neighborhood environmental exposures → health behavior outcomes.

The latter (neighborhood environmental exposures → health behavior outcomes) could be derived from extensive literature on built environmental health [18,19,20,21], consistent with the classical Bronfenbrenner’s socio-ecological model, which made environmental-level health interventions popular [22]. Specific to obesity-related environmental intervention research, the improvement of food and physical activity environments has been proven to contribute to healthier eating and physical activity behaviors by a large number of studies [23,24,25]. Sports facility density was found to be related to physical activity behavior, and greenery correlated with sedentary behavior [26,27]. The local retail food environment was also found to be related with dietary behaviors in Hong Kong by one of our former studies [28]. Therefore, the food and physical activity environment in terms of the relative density of fast-food restaurants and convenience stores compared to grocery stores, sports facility density, and street greenery were included in this study [26,28].

Next, we state the theoretical foundation and the logical inference of the former part (PRH residence → neighborhood environmental exposures) and the related hypotheses (PRH residence → unhealthy food environment/higher sports facility density/less street greenery) in the context of Hong Kong. Many studies have explored the environmental determinants of residential satisfaction with PRH in many cities [29,30,31]. However, studies on systematic disparities of residential environment in PRH and non-PRH are limited despite the stereotype of PRH being associated with poor living conditions. In Hong Kong, one study reveals that people living in public and private housing have significantly different built environments such as population density, the percentage of people living and working in the same district, PRH density, and a gravity-based accessibility [32]. Another study also found a significant difference in the satisfaction levels of the living environment, environmental quality, security, and urban renewal among residents from different housing types in Hong Kong [33]. These results follow the concept of “housing classes” developed by Rex and Moore (1967) that proposes different housing types are characterized by different physical and social settings [33,34]. PRH and non-PRH often distribute different places with their own neighborhood characteristics. Additionally, from the perspective of governance disparities, PRH in Hong Kong, as well as facilities and services in PRH communities, were provided and managed by the Hong Kong Housing Authority under the Hong Kong Government, while those in private housing were provided by private companies. Therefore, in this study, we assumed that PRH communities had different neighborhood food and physical activity environments than non-PRH communities. Specifically, PRH was assumed to be related to poor food and physical environments in terms of a higher relative density of fast-food restaurants and convenience stores compared to grocery stores, less sports facility density, and less street greenery.

## 3. Materials and Methods

### 3.1. Study Area

This study was conducted in Hong Kong, a special administrative region of the People’s Republic of China. As one of the most densely populated areas in the world, Hong Kong covers approximately 1080 square kilometers of land and had a population of 7,336,585 people at the end of June 2016, with 7,116,829 usual residents and 219,756 mobile residents [14]. Hong Kong is one of the cities with the highest inequality in the world, with an income Gini coefficient of 0.524 in 2016, and 1 in 5 people lived below the poverty line in 2017 [14]. Approximately 2.13 million persons (29.1%) lived in PRH, 1.16 million persons (15.8%) lived in subsidized home ownership housing, and 3.9 million persons (53.2%) lived in private permanent housing with a median monthly mortgage payment of HKD 10,500 (Hong Kong Dollar) [14].

The housing problem is worsened due to a lack of housing land, and Hong Kong has been ranked the least housing-affordable city in the world for a decade since 2011 by an international housing organization, Demographia [35]. An insufficient supply of land also leads to inadequate sites for community facilities. Even after planning for land supply, Hong Kong will still face a land shortage of at least 1200 hectares by 2046 [36]. In 2014, the Government of Hong Kong Special Administrative Region adopted a supply-led housing strategy with a commitment to continuously increase the housing supply, which strengthens the role of public housing and keeps an interchangeable ratio between PRH and subsidized sale flats. As a part of its long-term housing strategy, it adopted a 60:40 public–private split for new housing from 2015–2016 to 2024–2025, with 280,000 public housing units (200,000 PRH and 80,000 subsidized sale flats). In 2018, it revised the public–private split to 70:30 for the next 10-year period due to the strong social demand of public housing [37].

### 3.2. Data Collection

We used a representative sample of 1977 Hong Kong adults (aged or over 18) derived from a two-stage stratified sampling design by geographical area and housing type. Survey data of the 1977 adults were part of the first wave of the Strategic Public Research (SPPR) project, “Trends and Implications of Poverty and Social Disadvantages in Hong Kong: A Multi-disciplinary and Longitudinal Study” (for methodology details, see references) [38,39]. This was conducted by the Department of Social Work, the Chinese University of Hong Kong. The Survey and Behavioral Research Ethics Committee of the Chinese University of Hong Kong reviewed and approved of the study procedures and materials. The detailed verbal and written information on the study was given to participants. Because of the statistical treatment of data, personal data arising from the study are kept in strictly confidential ways. This sample has detailed information on the latitude and longitude of the respondents’ homes, and the cross-sectional survey data were acquired via face-to-face interviews conducted between May 2014 and July 2015. In this study, the following variables were collected: individual variables, such as housing type, sociodemographic variables, and variables of health behaviors, and perceived environmental variables.

Multi-source objective environmental data were also collected in this paper. To measure the local retail food environment, data on different food outlets were collected from Hong Kong’s Food and Environmental Hygiene Department [28]. To measure physical activity environment, data on sports facilities were collected from Hong Kong’s Leisure and Cultural Service Department. Data on street greenery were collected from Google Street View Images, using an object-based images classification algorithm. All objective environmental measures in this paper were constructed within a buffer around each respondent’s residence, using the Geographic Information System (GIS).

### 3.3. Independent Variable

The independent variable is PRH residence. Housing type of PRH was coded as 1 while housing type of non-PRH including subsidized sale flats and private housing was coded as 0.

### 3.4. Mediating Variables

Three measures of environmental exposures (Retail Food Environment Index (RFEI), sports facility density, and street view greenness (SVG)) were used as potential mediators. ArcGIS 10.4.1 was used to construct objective environmental measures. The objective environmental measures were standardized with a mean of 0 and a standard deviation of 1. We defined each respondent’s neighborhood as the area within a 1 km of Euclidean circular buffer from their home, which was considered as a walkable distance. RFEI was adopted from our previous study to characterize the unhealthy food environment [28]. It was calculated based on the relative density of fast-food restaurants and convenience stores to grocery stores. A higher RFEI indicates an unhealthier food environment.

For characterizing physical activity environment, we used sports facility density and SVG. Data on sports facilities were collected from the Leisure and Cultural Service Department, Hong Kong (https://www.lcsd.gov.hk (accessed on 12 December 2021). Sports facilities were defined as sports center, recreation grounds, water sports centers, swimming pools, or sports grounds [26]. We did not use individually listed sports facilities because they are provided by relevant place. The addresses of the public sports facilities were geocoded to obtain the latitude and longitude using Google Maps application programming interface (API). The density of public sports facilities was calculated in ArcGIS 10.4.1 using the Buffer and the Count Points in Polygon analysis tools.

Street greenery was characterized by SVG-derived street view images using an object-based images classification algorithm. For measuring SVG, we requested a series of images using the Google Street View application programming interface. Referring to the method used in previous studies, the sampling points along the road network were at 100-m intervals, and images were taken in 6 main directions (i.e., 0°, 60°, 120°, 180°, 240°, and 300°) [40,41]. The images were downloaded at each sampling point to cover the full horizontal field of view. To reduce computational time and to consider the actual needs of this study, we only used sampling points that fell within the respondent’s neighborhood (i.e., a 1-km Euclidean distance around their home). We obtained 48,250 street-view images in total. We used the object-based image classification algorithm pymeanshift to classify greenery in the images [40]. We calculated the average percentage of greenery in street-view images in six horizontal directions at the sample site and averaged greenness within the neighborhood to represent the SVG of each respondent. We used modified Python scripts based on an open source python library (https://github.com/mittrees/Treepedia_Public (accessed on 24 May 2020).

### 3.5. Dependent Variables

Three variables of obesogenic behaviors, including infrequent FV eating, physical inactivity, and prolonged sitting, were constructed as outcomes. Infrequent FV eating was defined as not having fruit or vegetables every day. FV consumption frequency data of respondents were collected in the cross-sectional survey. Respondents were asked “How many days a week do you usually have: (1) fruits and (2) vegetables”. Response options were continuous number from 1 to 7 (days). For analysis here, we dichotomized the outcome: infrequent FV eating (<7 days/week) and frequent FV eating (=7 days/week) since daily FV consumption is recommended by WHO.

Physical inactivity was defined as lack of vigorous physical activities such as heavy lifting, digging, aerobics, or fast bicycling for at least 10 min at a time during the last week and lack of moderate physical activities such as carrying light loads, bicycling at a regular pace, or doubles tennis during the last week (do not include walking). Prolonged sitting was defined as over six hours of sitting on a weekday. Physical inactivity and prolonged sitting were assessed by the International Physical Activity Questionnaire short form (IPAQ short) [39]. They were dummy variables based on the responses to the following questions: “During the past week, did you do vigorous physical activities such as heavy lifting, digging, aerobics, or fast bicycling for at least 10 min at a time? and did you do moderate physical activities, not including walking, such as carrying light loads, bicycling at a regular pace, or playing doubles tennis?”, and “During the past week, did you spend longer than 6 h sitting on a weekday?”. Affirmative responses were coded as 1.

### 3.6. Covariates

Socio-demographic variables and perceived environmental variables extracted from the cross-sectional survey data were covariates, including age, gender, marital status, subjective poverty (“Do you think you are poor now”), and monthly income (equivalized household income; this study used an equivalence scale which divided household income by square root of household size) [38], educational attainment, birthplace, having under-school-aged children, being a Hong Kong permanent resident, area problems, and accommodation problems.

Area problems and accommodation problems were used to characterize the perceived community and housing environment. To measure perceived community environment, residents were asked 12 items in relation to the question “Do you think that any of the things are a problem in this area?”: poor street lighting or broken pavement; noise (e.g., traffic, businesses); air pollution; lack of open public spaces; risk from traffic for pedestrians and cyclists; illegal parking; people being drunk or rowdy in the street/park; criminal activity; problems with communal areas (e.g., rubbish in corridors); rats or insects; and others (please specify). To measure perceived housing environment, residents were asked 12 items following the question “Do you have any of these problems with your accommodation?”: shortage of space; lack of privacy (within the household or between neighbors); too dark; not enough light; too hot in summer/too cold in winter; damp walls, ceilings, floors, etc.; poor ventilation; rats or insects; light pollution (too bright in the evening); and others (please specify). The number of area problems and the number of accommodation problems were summed for respondents.

### 3.7. Statistical Analyses

This study investigates the associations among PRH, food and physical activity environments, and obesogenic behaviors among adults in Hong Kong. As the objective environmental variables are continuous variables (Z-score) and the outcomes of interest are binary variables, we used the maximum likelihood method to estimate the parameters of our regression models. In addition, logistic regression was used since it is a useful analysis method for classification problems, and it often models a binary outcome or other two possible discrete outcomes. The statistical analyses were conducted using Mplus Version 8.3 (1998–2017 Muthen & Muthen). The statistical tests were two-tailed, with a significance level of 0.05. Unstandardized regression coefficients (B), standard errors (SE), 95% confidence intervals (CIs), and probability (P) were obtained.

First, to test the associations of PRH with food and physical environment, we regressed RFEI, sports facility density, and SVG on PRH, controlling for socio-demographic and perceived environment covariates.

Second, to test direct and indirect effects from PRH to obesogenic behaviors via food and physical environment, mediation analyses were conducted with several regressions based on the conceptual framework we developed in Figure 1. Three pathways were tested separately: (1) PRH residence → RFEI → infrequent FV eating; (2) PRH residence → sports facility density → physical inactivity; and (3) PRH residence → SVG → prolonged sitting. All the mediation models were adjusted for significant socio-demographic and perceived neighborhood and housing environmental covariates. The Hosmer and Lemeshow test (*p*) was used to test the fitting degree of the models. If *p* > 0.05, the fit of the model is good. The direct and indirect effects via corresponding food and physical environments were calculated. The proportion of the effect that is mediated was calculated as indirect effect/(direct effect + indirect effect) and presented as a percentage. The Sobel test was applied to test whether the indirect effect was significant.

## 4. Results

### 4.1. Decriptive Statistics

Table 1 shows the sample characteristics. Over half (57.1%) of the sample lived in PRH. This sample included 477 (24.1%) young adults (aged 18–39), 804 (40.7%) middle-aged persons (aged 40–59), and 696 (35.2%) older persons (aged 60 or over). Over half of the 1977 adults (51.6%) were not born in Hong Kong, but 95.1% of the 1977 adults had permanent Hong Kong residence. Over half (58.9%) of the participants were female, 24.1% were in subjective poverty, and 26.7% had equivalized household monthly income under HKD 3500. Among the respondents, 85.4% had not attended university, 38.2% were unmarried, and 91.7% did not have under-school-aged children.

Table 2 shows descriptive statistics of the objective environmental characteristics and obesogenic behaviors among adults with PRH and non-PRH residence. PRH residence had higher averages of sports facility density, but higher RFEI and less SVG than non-PRH residence, indicating better sports environment and unhealthier food environment and worse street greenery environment in PRH communities.

Non-parametric tests among PRH and non-PRH residences for environmental exposures and obesogenic behaviors were conducted to compare their distributions. Bold numbers indicate significantly different distributions with the non-parametric test (*p* < 0.05). The food and physical activity environment of PRH residence is significantly different from non-PRH residence. The obesogenic behaviors of infrequent FV eating and physical inactivity among residents in PRH are significantly different from those in non-PRH. Specifically, residents in PRH had significantly higher rates of infrequent FV eating and lower rates of physical inactivity than those in non-PRH.

### 4.2. Results of Regressions

Table 3 shows the associations of PRH residence with food, sports, and greenery environments. PRH residence was significantly associated with RFEI, sports facility density, and SVG, indicating that people living in PRH had very different food and physical activity environments from those living in non-PRH. Specifically, PRH residence was positively associated with RFEI and sports facility density and negatively associated with SVG, suggesting that PRH residence provided a better sports environment, but an unhealthier food environment and less greenery than non-PRH residence.

### 4.3. Results of Mediation Models

Table 4 shows the results of mediation models, which estimate the direct and indirect effects from PRH residence to obesogenic behaviors via food and physical activity environment, controlling for socio-demographic and perceived environmental variables. For the direct associations from PRH to obesogenic behaviors, PRH residence showed significantly negative association with physical inactivity but significantly positive association with infrequent FV consumption among adults in Hong Kong. For the indirect associations via food and physical activity environments, PRH residence showed a positive indirect association with infrequent FV eating via RFEI, a negative indirect association with physical inactivity, and a positive indirect association with prolonged sitting. The findings suggested that PRH residence was negatively associated with physical inactivity directly, as well as indirectly, via better sports environment. However, PRH residence was positively associated with unhealthy diet largely directly and positively associated with prolonged sitting indirectly via worse greenery environments.

## 5. Discussion

To the best of our knowledge, this is the first study to examine the associations between PRH and obesogenic behaviors as well as the mediator role of food and physical activity environment. It was conducted in the world’s least housing-affordable city with the data from a representative sample of residents aged 18 or over. Overall, PRH residence was positively associated with infrequent FV eating and negatively associated with physical inactivity among adults in Hong Kong, and these associations were partially mediated by worse neighborhood food environments and better sports environments, separately. The empirical findings in the paper also provide local governments implications for creating healthy residential environments for PRH residents and emphasizing the importance of neighborhood environment in mitigating health disparities induced by housing type. Below, we discuss and explain the results in more details.

Firstly, we found that residents in PRH had significantly higher rates of infrequent FV eating and lower rates of physical inactivity than those in non-PRH. Residing in PRH was not directly associated prolonged sitting. Our results also partly explain the mixed results of previous literature on the association between PRH residence and obesity [9,12]. They were possibly caused by the competition of the two forces, including eating and physical activity behaviors. From the current evidence, it is difficult to extrapolate the association between housing type and obesity in Hong Kong, since PRH residents showed unhealthier eating behavior on the one hand and healthier physical activity behavior on the other hand. The lower rate of physical inactivity in PRH residents could be a breakthrough in improving the health of low-income households and alleviating health disparities.

Secondly, contrary to our preconceived assumption, PRH was positively associated with higher sports facilities density. This result also explained the unexpected significantly lower rate of physical inactivity in PRH residence in Hong Kong. Although the result was surprising at first glance, it was a happy ending and could be explained by the logic behind sports facility planning in Hong Kong. Different from the local retail food environment, which was dominated by the capital market, public sports facilities were provided and distributed by the government. The Planning Department’s Hong Kong Planning Standards and Guidelines (2021) list three types of sports facilities including regional, district, and local sports facilities [42]. Local sports facilities in PRH communities were provided by the Housing Authority and were improved timely by “Estate Improvement Program”. In addition, the Housing Authority especially provides sports facilities suitable for older persons in PRH due to the concentration of elderly persons in PRH communities caused by the older-person-first PRH allocation policy. Furthermore, while other regional and district sports facilities provided by the Leisure and Cultural Service Department were planned based on population density, PRH in Hong Kong was proved to be associated with higher population density [32]. Therefore, PRH residents have more accesses to sports facilities which could be the reason leading to lower rate of physical inactivity than non-PRH residents.

Thirdly, PRH was positively associated with unhealthier food environment and negatively associated with street greenery indicating a worse food and greenery environment in PRH, which partially explained the significantly higher rate of infrequent FV eating and fully explained the non-significant but higher rate of prolonged sitting in PRH residents. These results also contribute the international review of PRH’s health impact and are consistent with the previously reported inverse association between PRH residence and poor health outcomes in developed Western societies and developed Asian countries such as Singapore [4,5,6]. Additionally, our results provide the cross-sectional evidence of environmental pathways linking PRH residence to potential health-related outcomes, such as obesity and chronic disease. These results correspond with another study conducted in Hong Kong, which indicated that positive associations between residing in PRH and premature mortality risk could be attenuated or even reversed after controlling for neighborhood characteristics [43]. Furthermore, we need to notice that only a small part of the association between PRH and infrequent FV eating could be explained by unhealthier GIS-based objective food environment. These results suggest that despite the objective food environment, there were other unshown factors that mediate the association between PRH residence and eating behaviors. A previous study summarized five dimensions of “food access”, including availability, accessibility, affordability, accommodation, and acceptability that affect residents’ eating behaviors [44]. Therefore, in addition to food accessibility, we need to pay more attention to other dimensions of “food access”, especially affordability. For example, the relative price of other foods to FV near the PRH residential areas could make a difference in eating FV behavior.

The findings in this study advanced international review in several ways including health impact assessment of PRH, environmental pathways linking PRH residence to health, and the association between residential environment and health outcomes. In addition, these findings contribute to the possible solutions of health disparities induced by housing type by improving neighborhood environment in health disadvantaged communities. Hong Kong has a rich experience in PRH governance. The PRH practice in Hong Kong provides a reference for other cities with housing problems. This is first study in Hong Kong linking PRH residence to health-related behaviors. More studies need to be conducted in Hong Kong and compared with other cities for creating a healthy and inclusive residential environment for low-income households.

This study also had several limitations. First, we were unable to establish a causal association between PRH residence, neighborhood environment, and behaviors due to the cross-sectional nature of the study. In addition, the mediation analysis we used comes with several assumptions and considerations about causal mechanisms that might bias the estimation of mediation effects when one or more assumptions are violated (e.g., exposure-induced mediator–outcome confounding) [45,46]. In future studies, we could make use of policy interventions to cleverly design experiments and use natural experiments to explore the causal relationship of housing and health. Second, we did not consider the subjective perceived factors of food and physical activity environments. Although we included perceived environmental covariates in this paper, the variables were rude and do not finely reflect the perceived food and physical activity environments. Further studies could be conducted to compare the difference of objective food and physical activity environment with perceived food and physical activity environment. Third, we need to note that only the density of sports facilities was considered. Even though there were more sports facilities positioned near PRH communities, the type of sports facilities could affect different sports behaviors. Lastly, the self-reported measures of behavior outcomes could be another limitation. More studies should be conducted to assess behaviors more comprehensively and objectively (e.g., accelerometry).

## 6. Conclusions

We found that residing in PRH was associated with higher rate of infrequent FV eating and lower rate of physical inactivity in the context of Hong Kong. The associations between residing in PRH and obesogenic behaviors were mediated by neighborhood food and physical activity environments. More targeted interventional studies directed at PRH communities need to be carried out to address the health inequalities induced by housing type and understand the causal associations among PRH, neighborhood environment, and health outcomes. These will aid policy makers in formulating better policies to improve health for low-income residents living in PRH and mitigate their health disparities with residents living private housing.

## Figures and Tables

**Table 1:** Characteristics of participants (*n* = 1977).

	Sample (*n* = 1977)
Independent variable: RRH residence	*n* (%)
Code = 1	1129 (57.1)
Code = 0	848 (42.9)
Mediating variables: food and physical activity environment	Mean (SD)
RFEI	0 (1)
Sports Facility Density	0 (1)
SVG	0 (1)
Dependent variables: obesogenic behaviors	%
Infrequent FV eating	36.1%
Physical Inactivity	36.8%
Prolonged Sitting	43.1%
Covariates	*n* (%)
Gender (*n* (%))	
Female	1164 (58.9)
Male	813 (41.1)
Age (*n* (%))	
18–39	477 (24.1)
40–59	804 (40.7)
60+	696 (35.2)
Monthly income (*n* (%))	
Not low income	1449 (73.3)
Low income (<HK$ 3500)	528 (26.7)
Self-reported poverty b (*n* (%))	
No	1500 (75.9)
Yes	477 (24.1)
Educational attainment (*n* (%))	
Higher education	289 (14.6)
Education attainment under college	1688 (85.4)
Marital status (*n* (%))	
Non-single	1221 (61.8)
Single	756 (38.2)
Birthplace (*n* (%))	
Non-Hong Kong	1020 (51.6)
Hong Kong	957 (48.4)
Having under-school-aged children (*n* (%))	
Yes	165 (8.3)
No	1812 (91.7)
Hong Kong permanent residence (*n* (%))	
Yes	1881 (95.1)
No	96 (4.9)
Area Problems (Mean (SD))	0.87 (1.277)
Accommodation Problems (Mean (SD))	1.30 (1.748)

Note: PRH = public rental housing; RFEI = Retail Food Environment Index; SVG = street-view greenness; FV = fruit and vegetable. Housing type of PRH was coded as 1 while housing type of non-PRH including subsidized sale flats and private housing was coded as 0.

**Table 2:** Descriptive statistics of environmental exposures and obesogenic behaviors among adults with PRH and non-PRH residence.

	PRH *n* = 1129	Non-PRH *n* = 848
Environmental Exposures	Mean (SD)	Mean (SD)
RFEI	**0.123 (1.091)**	**−0.163 (0.836)**
Sports Facilities Density	**0.135 (1.022)**	**−0.180 (0.941)**
SVG	**−0.073 (0.981)**	**0.097 (1.017)**
Obesogenic behaviors	Positive Rate (%)	
Infrequent FV eating	**40.1%**	**30.8%**
Physical inactivity	**32.9%**	**42.1%**
Prolonged sitting	43.3%	42.9%

Note: PRH = public rental housing; RFEI = Retail Food Environment Index; SVG = street-view greenness. Bold number indicating significant different distributions with non-parametric test (*p* < 0.05). Positive rate was calculated by the proportion of affirmative response.

**Table 3:** Regression model results of RPH residence on food, sports, and greenery environment.

Independent Variable: PRH Residence	B (S.E.)
Dependent variable: RFEI	0.268 (0.048) ***
Dependent variable: Sports facility density	0.314 (0.047) ***
Dependent variable: SVG	−0.161 (0.048) ***

Notes: *** *p* < 0.001; PRH = public rental housing; RFEI = Retail Food Environment Index; SVG = street-view greenness. Models adjusted for significant socio-demographic and perceived environmental covariates.

**Table 4:** Mediation results of direct and indirect effect from PRH residence to obesogenic behaviors via food environment and physical activity environment among adults in Hong Kong.

Independent Variable: PRH	Direct Effect	Indirect Effect	Hosmer and Lemeshow Test	Mediation Proportion
	B (S.E.)	B (S.E.)	(*p*)	(%)
Direct effect on infrequent FV eating	0.432 (0.112) ***			
Indirect effect via RFEI		0.030 (0.015) *	> 0.05	6.49
Direct effect on physical inactivity	−0.417 (0.124) ***			
Indirect effect via sports facilities density		−0.213 (0.040) ***	> 0.05	33.81
Direct effect on prolonged sitting	0.038 (0.105)			
Indirect effect via SVG		0.043 (0.016) **	> 0.05	53.09

Notes: * *p* < 0.05, ** *p* < 0.01, *** *p* < 0.001; PRH = public rental housing; RFEI = Retail Food Environment Index; SVG = street-view greenness; FV = fruit and vegetables. The proportion of the effect that is mediated (mediation proportion) was calculated as indirect effect/(Direct effect + indirect effect), and presented as percentage. Mediation models were adjusted for significant socio-demographic and perceived environmental covariates. The Sobel test was applied to test whether the indirect effect was significant. Hosmer and Lemeshow test (*p*) was used to test the fitting degree of the models. If *p* > 0.05, the fit of the model is good.

## Data Availability

Not publicly available.
